# Influencing factors on the NMP-22 urine assay: an experimental model

**DOI:** 10.1186/1471-2490-12-23

**Published:** 2012-08-28

**Authors:** Makito Miyake, Steve Goodison, Evan Gomes Giacoia, Wasia Rizwani, Shanti Ross, Charles J Rosser

**Affiliations:** 1 Cancer Research Institute, MD Anderson Cancer Center Orlando, Orlando, FL, 32827, USA; 2 Section of Urologic Oncology, MD Anderson Cancer Center Orlando, 1400 S. Orange Ave, Orlando, FL, 32806, USA

**Keywords:** Bladder cancer, Urine, NMP-22

## Abstract

**Background:**

The commercial NMP-22 urine assays for bladder cancer (BCa) detect nuclear mitotic apparatus protein 1 (NUMA1) using monoclonal antibodies. It remains unclear whether these assays are monitoring a tumor antigen or some other phenomenon associated with the disease state. In this study, we investigated the influence of urinary cellular and protein concentration, and hematuria on the performance of the NMP-22 tests in an experimental model.

**Methods:**

Pooled urine from healthy subjects were spiked with varying concentrations of benign (UROtsa) cells, cancer cells (RT4, T24, KU-7 and UM-UC-14), whole blood or serum, prior to analysis with both NMP22® Bladder Cancer ELISA test and the NMP22® BladderChek® point-of-care test.

**Results:**

Urines from control subjects were negative for NMP-22. The addition of whole blood at 50ul/10 ml, but not serum, resulted in a false-positive result. Furthermore, the addition of a high concentration of benign urothelial cells (10^6^) or the cell lysate from these cells (306 μg protein) resulted in a false-positive result. High concentrations of pooled-cancer cells (10^6^) or cell lysate (30.6 μg and above) resulted in a positive NMP-22 assay. Concordance between the NMP-22 ELISA assay and the NMP-22 point of care assay was >90%.

**Conclusions:**

Rather than detecting a specific tumor antigen, urinary NMP-22 assays may be measuring the cellularity or amount of cell turnover that may be introduced into the urine by a variety of conditions, including surface shedding from bladder tumors. The absence of significant urinary cellularity in some cases due to lesion characteristics or the timing of sampling may result in false-negative NMP-2 assays.

## Background

Non-invasive urine tests for the early detection or post-surgical surveillance of bladder cancer (BCa) are highly desirable for both patient and healthcare system. Currently, voided urinary cytology (VUC) is the most widely used non-invasive urine test, with reported sensitivities ranging from 13–75% and specificities ranging from 85–100% [1,2]. A couple of single-biomarker urinalysis assays have been developed for use in this context. One is the bladder tumor antigen (BTA) test, which detects urinary complement factor H-related proteins using monoclonal antibodies [3]. Two assay formats, BTA stat™ and BTA TRAK™ (Polymedco Inc. Cortlandt Manor, NY, USA), are FDA approved for the detection and surveillance of BCa in urine samples. For BCa detection, urinary BTA tests have diagnostic sensitivities ranging from 29–83% and specificities ranging from 56–86% [4,5]. Recently, Oge *et al.* demonstrated that hematuria adversely affects the accuracy of BTA assays [6].

Another commercial assay that has been used for BCa detection in voided urine samples is the nuclear matrix protein-22 (NMP-22) test, which detects the nuclear mitotic apparatus protein 1 (NUMA1), using monoclonal antibodies [7]. Two assay formats, the NMP22® Bladder Cancer ELISA Test Kit and the NMP22® BladderChek® point-of-care test (Alere Scarborough, Inc. Waltham, MA), are FDA approved for the detection and surveillance of BCa in urine samples. For BCa detection, urinary NMP-22 tests have diagnostic sensitivities ranging from 47–100% and specificities ranging from 55–98% [8,9]. It remains unclear whether the NMP-22 test is monitoring the increased expression of a *bona fide* tumor antigen, or some other phenomenon associated with BCa such as hematuria or urothelial turnover. Noting the limitations of the BTA assays, we investigated the influence of urinary cellular concentration, urinary protein concentration and hematuria on the performance of the NMP-22 tests in an experimental model.

## Methods

### Clinical sampling and processing

Under IRB approval and informed consent, serum samples were collected from 20 men with no previous history of BCa and 20 men with newly diagnosed BCa. Serum was isolated, snap frozen and stored at -80^0^C until use. Two hundred ml of urine and 10 ml of whole blood were collected from three healthy subjects. None of the subjects had any current or history of urological disorder. Microscopic examination of the urine samples showed no cells and all samples were tested by urinary dipstick test for abnormalities. Pertinent clinical information for each subject was recorded.

### Cell lines and culture

Human bladder cancer cell lines; RT4 (ATCC, Manassas, VA), T24 (ATCC, Manassas, VA), UM-UC-14 (a generous gift from Dr. H. Bart Grossman, The University of Texas M.D. Anderson Cancer Center, Houston, TX), and KU-7 (a generous gift from Dr. Motoyoshi Tanaka, Nara University Medical School, Nara, Japan) were available for analysis. The benign human bladder cell line, UROtsa, was a generous gift from Dr. Donald Sens at the University Of North Dakota School Of Medicine (Grand Forks, ND). T24, UM-UC-14 and KU-7 cell lines were maintained in RPMI1640 media. UROtsa and RT4 cells were maintained in low-glucose DMEM and McCoy’s 5A medium (Life Technologies, Inc., Gaithersburg, MD), respectively. All media were supplemented with 10% fetal bovine serum, 100 units/ml of penicillin and 100 μg/ml of streptomycin. All cells were incubated at 37°C in a humidified atmosphere of 5% CO_2_ in air.

### Experimental model

**Figure 1:**
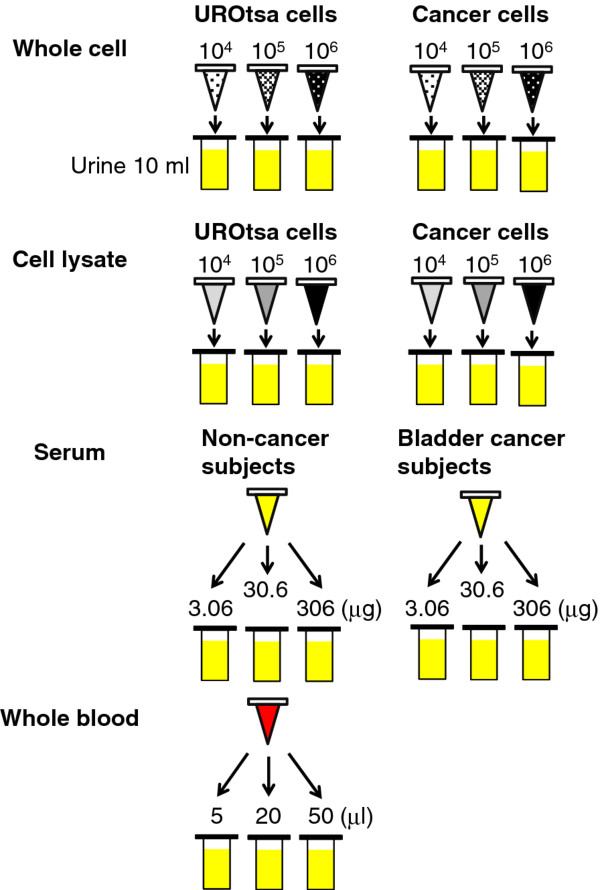
**Schematic of experimental model.** Low concentration (1×10^4^), medium concentration (1× 10^5^) and high concentrations (1× 10^6^) of UROtsa benign human bladder cells, or a mixture of human bladder cancer lines, RT4, T24, UC-UM-14 and KU-7 were added to 10 ml of pooled urine from three healthy controls. The cellular lysate associated with low protein concentration (3.06 μg), medium concentration (30.6 μg) and high concentrations (306 μg) of UROtsa benign human bladder cells or a mixture of human bladder cancer lines, RT4, T24, UC-UM-14 and KU-7 were added to 10 ml of pooled urine from three healthy controls. In addition, whole blood (1, 5, 20 and 50 μl) and serum (3.06, 30.6 and 306 μg protein) were added to 10 ml of pooled urine from healthy controls.

To create the experiment model, 200 ml of freshly voided urine samples from three healthy controls were collected in sterile containers. All subjects’ urines tested negative for NMP-22 using both the NMP22® Bladderchek® test and the NMP22® ELISA kit assay. The urine samples from the three subjects were pooled, mixed and distributed into 10 ml aliquots in 15 ml centrifuge tubes. The human bladder cell lines were washed, trypsinized and counted. For UROtsa, 1 ×10^4^ (low concentration), 1 ×10^5^ (medium concentration) and 1 ×10^6^ (high concentration) cells were each added to triplicate 10 ml pooled urine samples. Equal numbers of RT4, T24, UM-UC-14 and KU-7 were pooled, and 1 ×10^4^ (low concentration), 1 × 10^5^ (medium concentration) and 1 × 10^6^ (high concentration) cells were added to triplicate 10 ml pooled urine aliquots. For cell lysate analyses, 1 × 10^6^ cells of each cell line were lysed by RIPA buffer (Pierce, Rockford, IL) and total protein concentration measured. The total proteins extracted from 1 × 10^6^ cells of UROtsa, RT4, T24, UM-UC-14 and KU-7 were 431ug, 369ug, 174ug, 261ug and 293 μg, respectively, with a mean total protein extract of 306 μg. In the following experiments, 3.06 μg, 30.6 μg and 306 μg of cellular proteins corresponding to 1 × 10^4^ (low concentration), 1 × 10^5^ (medium concentration) and 1 ×10^6^ (high concentration) cells, were used. These UROtsa lysates and pooled cancer cell lysates were added to pooled urine samples in triplicate. To monitor hematuria influence, pooled whole blood (1, 5, 20 and 50 μl) from 20 subjects with and 20 subjects without BCa was added analyzed in triplicate in 10 ml urine samples. Serum samples from 20 subjects with and 20 subjects without BCa were pooled. Serum protein concentrations similar to the cellular lysate concentrations, 3.06 μg (low concentration), 30.6 μg (medium concentration) and 306 μg (high concentration) were added to triplicate pooled urine samples. Figure 1 illustrates the experimental model.

### Reagents and tests

The NMP-22 assays: NMP22®BladderChek Test® and NMP22® Bladder Cancer ELISA Test Kit were purchased from Alere Scarborough, Inc. (Waltham, MA). The NMP22®Bladderchek Test® was performed by adding 4 drops of urine into the well of a disposable test device for all urine samples. Results were considered to be positive if any band, no matter how faint, appeared in the test zone 30 min after adding the urine to the well. The NMP-22 ELISA assay was conducted according to the manufacturer’s instructions. Briefly, 200 μl of urine was added to the 96-well plate. Calibration curves were prepared using purified standards for each protein assessed. Curve fitting was accomplished by linear regression following manufacturer’s instructions. Based on published literature, a cutoff value of 10 U/mL was used to define a positive NMP-22 ELISA assay [8,10]. Urinalysis was performed with MULTISTIX PRO Reagent Strips (Bayer HealthCare, Elkhart, IN) to assess protein. In addition, urinary protein concentration was determined using Pierce 660-nm Protein Assay Kit (Thermo Fisher Scientific Inc., Waltham, MA, USA).

### Data analysis

A Student *t*-test was used to compare NMP-22 expression levels in cell lines. Statistical significance in this study was set at *p* < 0.05 and all reported *p* values were 2-sided. All analyses were performed with PRISM software version 5.00 (GraphPad Software, San Diego, CA).

## Results

**Figure 2:**
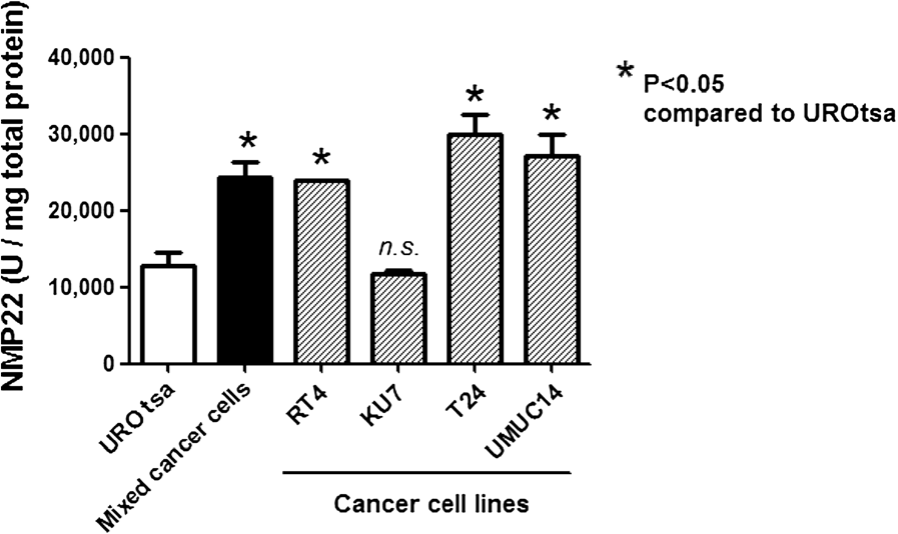
**NMP-22 ELISA assay of human bladder cell lines.** The human bladder cancer cell lines, RT4, T24 and UM-UC-14 had elevated NMP-22 levels compared to the benign UROtsa human cell line. Levels of NMP-22 in the KU-7 human bladder cancer cell line were not elevated compared to UROtsa. Significance (*p* < 0.05) was assessed by Student *t* test. *n.s.*, not significant.

The NMP22® Bladder Cancer ELISA Test was performed on a panel of human bladder cell lines (Figure 2). The cancer cell lines, RT4, T24 and UM-UC-14, had significantly higher NMP-22 levels than the benign cell line, UROtsa (*p* < 0.05). One bladder cancer cell line, KU-7, did not express higher NMP-22 levels than UROtsa. The NMP-22 level recorded for a pool of the four cancer cell lines was significantly higher than the NMP-22 level of UROtsa (24,299 ± 2,110 U/mg vs. 12,641 ± 1,973 U/mg, *p* < 0.05).

**Figure 3:**
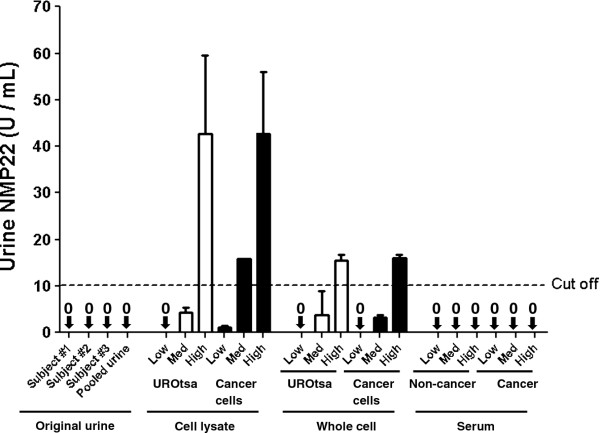
**NMP-22 ELISA assay of the experimental model.** Positive NMP assays were elicited by high concentrations of UROtsa cells, pooled cancer cells, cell lysate from UROtsa, and medium and high concentrations of cell lysate from pooled cancer cells. Mean levels are depicted by long horizontal lines. Error bars indicate standard deviations.

The results from applying both the NMP22® Bladder Cancer ELISA Test and the NMP22® Bladdercheck® Test on samples taken from our experimental model are described in Table 1 and Figure 3. Urine samples from Individual subjects, and a pool of those samples were negative for NMP-22. Whole cells or cell lysates of the cancer cell line pool and UROtsa cells were added to the pooled urine sample and re-analyzed using both NMP-22 ELISA tests. Only the high concentration (1 × 10^6^) of UROtsa or pooled cancer cells produced a positive NMP-22 test. Cellular lysates from 1 × 10^5^ and 1 × 10^6^ pooled cancer cells, and from 1 × 10^6^ UROtsa cells produced a positive result. The only discordance seen between the NMP22® Bladder Cancer ELISA Test and the NMP22® Bladdercheck® test was seen when UROtsa lysate (1 × 10^5^ cells) produced a positive NMP22® Bladdercheck® test but a negative NMP-22 ELISA assay.

**Table 1:** Results of NMP22® Bladdercheck® Test

Whole cells*		**Assay results**
**UROtsa**		
	10^4^cells	-
	10^5^cells	-
	10^6^cells	+
**Pooled cancer cells**		
	10^4^cells	-
	10^5^cells	-
	10^6^cells	+
Cell Lysate*		
**UROtsa**		
	3.06 μg	_
	30.6 μg	-
	306 μg	+
**Pooled cancer cells**		
	3.06 μg	-
	30.6 μg	+
	306 μg	+
Serum*		
**BCa**		
	3.06 μg	-
	30.6 μg	-
	306 μg	-
**Non-BCa**		
	3.06 μg	-
	30.6 μg	-
	306 μg	-
Whole blood*		
**BCa**		
	5 μl	-
	20 μl	-
	50 μl	+
**Non-BCa**		
	5 μl	-
	20 μl	-
	50 μl	+

*, added to 10 mL of pooled urine from healthy controls and analyzed; BCa, Bladder cancer.

The addition of whole blood at 5ul and 20ul per 10 ml of urine did not cause a positive NMP-22 result, but spiking 50 μl/10 ml did produce false-positive NMP-22 assays. The fact that the false-positive effect was cellular was confirmed by the addition of pooled serum samples obtained from subjects with or without BCa. Neither serum sample caused NMP-22 test positivity, even at the highest level of 306 ug of protein. Overall, the concordance rates between the NMP22® Bladdercheck® test and the NMP22® ELISA kit assay was > 90% throughout the analyses.

## Discussion

Using a novel experimental model, we evaluated a number of factors that may influence the NMP-22 urine tests. Although the model does not mimic the actual physiological situation exactly, the aim was to test whether the NMP-22 analyte could originate from blood (serum or whole blood) or benign cells of urothelial origin. Previous studies have investigated the influence of blood in the NMP-22 assay. Atsu *et al.* reported that when whole blood was added to a urine sample, positive NMP-22 results increased in parallel with the increase in the amount of red blood cells in the sediment. In addition, the leukocyte count in the urine sediment also had a significant impact, and the investigators concluded that pyuria and hematuria significantly affected urinary NMP-22 [11]. In our study, the influence of blood was confirmed, but we also showed that spiking with intact, benign or cancerous cells caused a positive NMP-22. As expected, cellular lysate from those cells had a greater effect.

**Figure 4:**
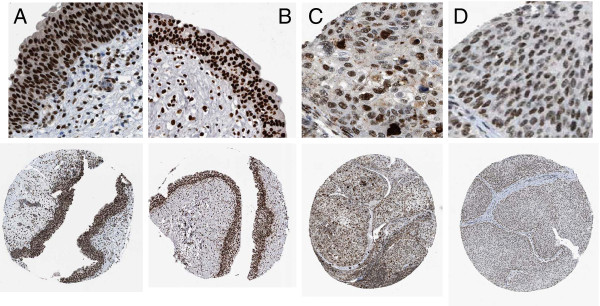
**Immunostaining of NMP-22 (NUMA1) in normal urothelia and bladder cancer tissue.** Images originate from the Human Protein Atlas web portal. Top panels show magnified insets of tissue microarray images directly below. **A**, normal urinary bladder tissue from a male of 37 years. **B**, normal tissue from a male of 51 years. **C**, bladder carcinoma tissue from a male of 51 years. **D**, bladder carcinoma tissue from a male of 78 years. The normal urothelial layer, and carcinoma tissues display strongly positive nuclear staining and less intense cytoplasmic staining.

NMP-22 (nuclear matrix protein-22) is also known as nuclear mitotic apparatus protein (NUMA or NUMA-1) [12,13]. The function of NUMA-1 is to provide structural support for the nucleus and to ensure the correct separation of genetic material during mitosis into the respective daughter cells through mitotic spindle stabilization [14]. To see how NUMA-1 is distributed in healthy and diseased tissues we queried the Human protein Atlas (http://www.proteinatlas.org). The Atlas provides immunohistochemistry data, and expression is confirmed in the majority of cases using 2 or more antibodies for the same antigen [15]. NUMA-1 expression data is available on a wide range of tissues, including both normal and diseased urinary bladder tissue. An overview of the staining patterns across tissues confirms that NUMA-1 is expressed in all tissues with an epithelial component, but also in glandular cells, cells of the lymph node and macrophages. Notably, it is apparent that the normal urothelial lining of the bladder expresses NUMA-1 at a high level (Figure 4). As reported previously, NUMA-1 is localized to the nucleus primarily, but there is granular cytoplasmic staining of NUMA-1 protein also. Carcinoma tissues typically have NUMA-1 expression across the tumor, with some expected heterogeneity, but intensity of staining is often less than that seen in the normal urothelia (Figure 4), and the cytoplasmic component is less pronounced. The imunohistochemistry data suggest that at the interface between urine and bladder lining, the cellular expression level will be at least as strong in normal tissue as in tumor tissue. 

We also queried public databases for transcriptome profile data. NUMA-1 expression is reported to be increased in tumors of a variety of tissues, but given the epithelial expression profile of NUMA-1 this can be misleading unless the details of how the cellular composition of solid tissue specimens was corrected for in specific studies are known. However, of note, the profile databases do report that NUMA-1 mRNA is high in whole blood. This is of interest because blood incursion into the bladder can create difficulties in interpretation of urine tests for bladder cancer. We showed in our experimental model that spiking of NMP-22 negative urine with whole blood can cause a false-positive result.

If the NMP-22 test is measuring a nuclear protein that is present in the majority of cell types throughout the body, and one that is particularly prevalent in the normal bladder lining, how does the test perform reasonably well for the detection of BCa. The test could be detecting NMP-22 that is introduced into the urine by hematuria, and since hematuria is the most associated symptom of bladder cancer, then the test would perform no better than a test for hemoglobin as long as the threshold and sensitivity of the two assays were comparable. Conversely, measuring NMP-22 introduced by bleeding would impact the sensitivity of the test, calling falsely positive cases that had hematuria through non-malignant causes such as infection or trauma. Alternatively, perhaps the NMP-22 test is a measure of the release and lysis of urothelial cells into the urine. Bladder tumors are often friable and shed large numbers of tumor cells into the urine relative to the normal turnover of healthy urothelia. The total number of urothelia shed per unit of time will be greater if the patient has a bladder lesion. NMP-22 is a nuclear scaffold protein and is not known to be secreted, thus, cell lysis must occur for the NMP-22 test to detect soluble protein. If cell lysis occurs at a constant rate, then the increased number of cells released into the urine in the presence of a lesion may suffice for a test, but it is also possible that tumor cells lyse in urine more readily than normal urothelia, thereby amplifying the test to some extent. A likely scenario is that a combination of these factors (more cells released and lysed, and the presence of blood) contribute to the NMP-22 test result. As long as the test outperforms VUC, one can argue that it has value, but identifying what any bladder cancer test is actually measuring would seem to be pertinent and should enable improvement of a given test, or provide insight into how best to combine multiple markers.

Other investigators have associated false-positive NMP-22 tests with instrumentation of the genitourinary tract and inflammation [16] and grade of urinary hematuria [17]. Specifically, Huber *et al.* reported from a large prospective screening study that NMP-22 outcomes are affected by gross hematuria, urinary tract infections and concentrated urine (creatinine > 2.5 g/L) [18]. Furthermore, impaired renal function (i.e., reduced glomerular filtration rate) has been linked to false-positive NMP-22 results, while proteinuria associated with impaired renal function is associated with reduction in urinary cytology specificity [19]. Similar to other urine-based assays, NMP-22 has a disappointingly low sensitivity for small, low-grade tumors [20]. Based on our results we believe the reduced sensitivity is related to low tumor burden and consequent low cell turnover. Perhaps if NMP-22 urinary protein was normalized to total urinary proteins, or to urinary creatinine, then a NMP-22 assay may be better able to diagnose these small, low-grade tumors, which are the most common, yet least threatening of the tumors.

## Conclusions

In combination, these findings raise the question of whether the NMP-22 tests are detecting a bona fide tumor antigen in urine samples or detecting a surrogate for cellularity/cell turnover or hematuria introduced into the urine by a friable bladder tumor. The absence of significant urinary cellularity in subjects with BCa may result in false-negative NMP-22 assays.

## Abbreviations

NMP-22: Nuclear matrix protein 22; NUMA1: Nuclear mitotic apparatus protein 1; BCa: Bladder cancer; VUC: Voided urinary cytology; BTA: Bladder tumor antigen; ELISA: Enzyme-linked immunosorbent assay.

## Competing interests

The authors declare that they have no competing interests.

## Authors’ contributions

MM Study concept and design, acquisition of data, data analysis, drafting of manuscript. SG Study concept and design, drafting of manuscript, supervision. EGG acquisition of data and data analysis. WR acquisition of data and data analysis. SR acquisition of data and data analysis. CJR Study concept and design, drafting of manuscript, supervision. All authors have read and approved the final manuscript.

## Pre-publication history

The pre-publication history for this paper can be accessed here:

http://www.biomedcentral.com/1471-2490/12/23/prepub
